# Difference in Estimation of Side Effects of Chemotherapy between Physicians and Patients with Early-Stage Breast Cancer: The Use of Patient Reported Outcomes (PROs) in the Evaluation of Toxicity in Everyday Clinical Practice

**DOI:** 10.3390/cancers13235922

**Published:** 2021-11-25

**Authors:** Mirjana Pavlović Mavić, Robert Šeparović, Ana Tečić Vuger, Ljubica Vazdar

**Affiliations:** 1 University Hospital Center “Sestre milosrdnice”, University Hospital for Tumors, 10000 Zagreb, Croatia or robert.separovic@unipu.hr (R.Š.); ana.tecic@kbcsm.hr (A.T.V.); ljubica.vazdar@kbcsm.hr (L.V.); 2 Medical School, Juraj Dobrila University of Pula, 52100 Pula, Croatia

**Keywords:** breast cancer, chemotherapy, toxicity, outcomes, physicians

## Abstract

**Simple Summary:**

Patient reported outcomes (PROs) are frequently integrated into routine toxicity monitoring in clinical studies but not so often in everyday clinical practice. Our investigation, conducted on patients with early stage breast cancer, showed disproportion between patient and physician perceptions of side effects by using a PRO questionnaire for patients and then the questionnaire with the same questions for physicians estimation. We found this result to be very important because the use of PRO in our clinical practice helped us determine the group of patients which required additional care to help them tolerate treatment-related side effects, have better quality of life during treatment and ultimately have best possible outcome.

**Abstract:**

Knowledge about the patient’s experience and perception of side effects and their impact on daily life is crucial for the adequate planning of interventions to provide the highest attainable levels of quality of life during oncology treatment. We conducted a study on consecutive samples of 69 early breast cancer patients treated with four cycles of neoadjuvant or adjuvant anthracycline-based chemotherapy. Patients completed the questionnaire about side effects experienced after the previous cycle of chemotherapy. The questionnaire was a modified PRO for the evaluation of treatment toxicity consisting of 18 questions related to the very common and common side effects of doxorubicin and cyclophosphamide, valued from 0 to 3 according to the subjective assessment of the patient. During the same cycles of therapy, data were also collected by the physician who completed a questionnaire consisting of the same questions as the questionnaire for patients, on the same scale. Most of the side effects reported by patients were mild to moderate in intensity, while physicians reported side effects much less frequently. The results also indicated a disproportionate reporting, in which physicians reported statistically significantly fewer side effects than patients. This study reported a level of disagreement between patients and physicians in the experience of therapy toxicity. In conclusion, use of PRO in clinical practice can help us avoid physician subjectiveness in the estimation of side effects and determine the group of patients who can benefit from additional and individualized supportive care measures, which could lead to better adherence to therapy and ultimately best outcomes.

## 1. Introduction

Introducing patients to the side effects of chemotherapy and its impact on quality of life (QoL) during treatment is an equally important part of communication between physicians and patients, as is discussing the prognosis of the disease itself.

Over the past few decades, several clinical trials have shown that the presence of physical symptoms and side effects can substantially affect patient acceptance and engagement in treatment, and thus indirectly affect treatment outcomes [1,2]. Over the last ten years, patient experiences of side effects and their impact on QoL and general well-being during systemic antineoplastic treatment, began to be taken into account and considered in treatment plans [3,4,5].

Patients perceive the content and adequacy of information obtained in a conversation with physician differently [6]. Accordingly, it is possible that the symptoms and side effects that occur during the course of treatment which are very important from the patient’s perspective, may be underestimated or misjudged by the physician designing the treatment. Even when verbal communication between physician and patient is adequate, an assessment of the intensity and duration of treatment side effects is often not recorded in the medical records of patients treated for early breast cancer [7].

The direct consequence of the need for better awareness of side effects is that the latest clinical studies include patients as equally valuable side in clinical studies. This is now a norm, and studies assessing toxicity and quality of life involve patients’ perspectives and input, most commonly in the form of patient reported outcomes (PROs). Given that such reports are relatively easy to implement in practice and offer valuable information, they are commonly included in daily clinical work.

Knowledge about the patient’s experience and perception of side effects and their impact on daily life is crucial for the adequate planning and implementation of interventions, with an aim to provide the highest attainable levels of quality of life during oncology treatment, and thus the best possible treatment outcome.

## 2. Materials and Methods

The study was performed on a consecutive sample of patients treated with neoadjuvant or adjuvant chemotherapy for early-stage breast cancer at the daily hospital of the Department of Medical Oncology of University hospital for Tumors in Zagreb between December 2018 and April 2019. All patients received four cycles of AC chemotherapy (doxorubicin + cyclophosphamide) before the surgery as part of a neoadjuvant treatment protocol, or after the surgery as part of an adjuvant treatment. All patients were administered a total dose of doxorubicin of 240 mg/m^2^ and of cyclophosphamide of 2400 mg/m^2^.

An approval for conducting the study was obtained from the Ethical committee of University Hospital Center “Sestre milosrdnice” Zagreb. All the methods were performed in accordance with the relevant guidelines and regulations, and all the patients gave their written informed consent prior to their inclusion in the study [8].

The study included 69 patients with a median age of 53 years (range 29–76 years). The characteristics of the patients included in the study are shown in Table 1. Treatment was discontinued in two patients; in one due to the development of cardiotoxicity and in the other due to evidence of metastatic disease during adjuvant treatment. One patient was excluded from the study due to a language barrier and the inability to answer the questions in the questionnaires independently. Seven patients did not complete all three planned questionnaires. A total of 196 sets of questionnaires (patient questionnaire + physician questionnaire) were available for analysis, 68 completed with cycle 2, 66 with cycle 3, and 62 with cycle 4.

### 2.1. Patient’s Reports of Side Effects

During the second, third and fourth cycle of chemotherapy, patients independently filled out a questionnaire about the side effects experienced after the previous cycle of chemotherapy (without the presence of a physician and other medical staff). The questionnaire was a modified PRO for the evaluation of treatment toxicity and it consisted of 18 questions related to the very common and common side effects of doxorubicin and cyclophosphamide (listed in the Summary of Product Characteristics). Side effects were valued from 0 to 3 according to the subjective assessment of the patient (0-no side effects, 1-mild, 2-moderate and 3-severe side effects).

The patients were asked about any other side effects experienced in the last question of the questionnaire. Answers to this question were descriptive and nonspecific, and the side effects that patients mentioned in this part of questionnaire were not valued so they were not included in the final statistical analysis.

### 2.2. Physician’s Reports of Side Effects

During the second, third and fourth cycles of chemotherapy, data were also collected by the same physician. The physician completed a questionnaire about the side effects consisting of the same questions as the questionnaire for patients, on the same scale (0-no side effect, 1-mild, 2-significant, and 3-severe side effect). The side effects were harmonized according to the CTCAE criteria [9]; if this was not possible, the assessment was based on physician’s estimation.

### 2.3. Data Analysis

The intensity of each side effect in the total study population was presented as the sum of the individual intensities of all possible side effects based on the patient’s assessment. The intensity of each side effect assessed by the physician was calculated in the same way. The analysis was performed by calculating the kappa coefficient, assessing the agreement between the patients and the physician. The values of the kappa coefficient up to 0.20 were considered low, values from 0.21–0.40 were considered mild, values from 0.41 to 0.60 were considered medium, and values 0.61 and higher were considered high coincidences. The chi square test was used in the analysis of categorical data. Data were analyzed by statistical programs Microsoft Excel 2019 (Microsoft, Redmond, WA, USA) and IBM SPSS (version 21.0, Chicago, IL, USA).

## 3. Results

Data for 69 pairs of patients and physician were analyzed. The highest intensities of side effects reported by patients were hair loss, fatigue and feeling of weakness, and nausea (Table 2). Kappa coefficients could be calculated for 39 out of 54 responses (physicians reported zero values in the rest, making it impossible to calculate kappa coefficients). The results had suggested 2 instances with high agreement, 11 with medium, 15 with mild match, and 11 instances of low agreement: notably, vomiting, watering and burning of the eyes had the best match (Table 2).

We also investigated the intensity of side effects across patients and physicians. Most of the side effects reported by patients were mild to moderate in intensity, while physicians reported side effects much less frequently (Table 3). There were strong statistically significant differences in the frequency of reporting between patients and physicians (*p* < 0.001 for all three cycles). A more detailed examination indicated a disproportionate reporting, in which physicians reported statistically significantly fewer side effects, both by individual side effect intensity and at the level of the entire sample (Table 3).

The intensity of side effects did not differ for patients between the second and third cycles (*p* = 0.138), or the second and fourth cycles (*p* = 0.341). However, physicians reported statistically significantly more side effects in the third and fourth cycles compared with the second (*p* = 0.008; *p* = 0.019).

## 4. Discussion

The discrepancy between patients and physicians experience of side effects is well known. In this study, we used this fact to objectify and emphasize the importance of use of PROs in everyday clinical practice by giving the same questionnaire to physicians and patients and showing that there is an objective need for this type of communication in order to overcome physician subjectiveness regarding side effects and their impact on patient quality of life.

Examining the overall results of the intensity of side effects reported by patients, it is evident that most of the side effects listed in the questionnaire were not reported at all, and the reported side effects were usually mild to moderate. This is in some way expected considering that the currently available symptomatic and supportive treatments have significantly improved the tolerability of chemotherapy. The intensity of side effects was estimated to be highest on the third measurement, i.e., after the second cycle of chemotherapy, which we attributed to the appearance of the most pronounced hair loss and increased fatigue in that time period. Other similar studies showed inconsistencies in the experience of certain side effects during treatment and concluded that assessments of side effects based on only one applied cycle can significantly underestimate the overall prevalence of a side effect throughout the entire course of treatment [10]. The side effects reported by the physician after talking to the patient were most often of mild intensity.

Analysis of the data obtained in our study showed that there was a statistically significant difference in the perception of the intensity of side effects reported by patients and physicians in the sense that physicians underestimate the intensity of symptoms experienced by patients. In several clinical studies, even those strictly controlled, it has been shown that physicians and nurses often underestimate the frequency and severity of symptoms compared to patient ratings [11,12,13]. One of the explanations offered is that patients do not report the actual extent of the problems they face in communication with the physician, unlike the assessments they state in self-completed questionnaires, because they believe that physicians will perceive their symptoms as insignificant [10]. There are several potential reasons for decreased perception of side effects by physicians. It is possible that physicians simply pay less attention and are less likely to report mild side effects and side effects that do not require the use of symptomatic therapy or a delay in the planned treatment cycle. Another proposed reason is the possible lower reporting of side effects that are common and expected with a particular chemotherapy protocol, and conversely, the lower reporting of very rare side effects that are sufficiently unexpected that physicians often forget to ask the patient about them [14].

The patient reported outcome (PRO) is by definition a measure of the patient’s condition reported directly by the patient without interference or interpretation by the clinician or anyone else, and over the past ten years such reports have begun to be integrated into routine toxicity monitoring and treatment side effects in clinical studies [15,16]. The World and European Medical Regulatory Agency (FDA and EMA) have published guidelines on the use of PROs in clinical studies, and the EMA has published guidelines on the use of PROs in clinical trials investigating antitumor drugs [17,18]. These facts clearly speaks to the importance of obtaining information from patients to assess the benefits and risks of various anticancer therapies, which is why PROs have today become one of the standard measures of treatment outcomes.

One of the main limitations of this study is a relatively small sample with a focus on only one diagnosis. The current condition of the patients could lead to problems when entering data, as daily changes could significantly affect perception. One of the methodological advantages is the use of repeated questionnaires, which increased the statistical power of the research and enabled the analysis of data over time.

## 5. Conclusions

The results of our study confirmed in clinical practice what had previously been shown in clinical trials, namely that the toxicity data reported by physicians were not consistent with the data reported by patients. In order to provide patients with the best possible quality of health care in terms of providing adequate and timely symptomatic and supportive care, and thus the best possible quality of life during treatment, it is necessary to take a step forward and implement outcome reports obtained directly from patients themselves.

The scope of this paper was to show how simple additional effort (use of PRO) in everyday clinical practice could give us real insight in patient’s experience and help us select the group of patients who might benefit from personalized supportive measures.

## Figures and Tables

**Table 1:** Patient characteristics (N = 69).

Characteristics		N	%
Localization	locoregional	37	53.6
	local	32	46.4
Biology	luminal A	2	2.9
	luminal B HER2 neg.	31	45.0
	luminal B HER2 pos.	23	33.3
	non-luminal HER2 pos.	6	8.7
	triple negative	7	10.1
Neoadjuvant therapy	no	31	44.9
	yes	38	55.1
Menopausal status	premenopause	36	52
	postmenopause	33	48

**Table 2:** Comparison of the intensity of reported side effects among patients and kappa coefficients for comparisons of the second, third and fourth treatment cycles.

Side Effects	Second Cycle			Third Cycle			Fourth Cycle		
	Patient’s Reports	Physician’s Reports	Kappa	Patient’s Reports	Physician’s Reports	Kappa	Patient’s Reports	Physician’s Reports	Kappa
Fatigue, feeling of weakness	116	72	0.271	132	88	0.238	130	90	0.232
Elevated temperature	22	9	0.258	18	8	0.185	18	1	0.048
Allergic reactions	6	0	-	7	0	-	8	1	0.026
Skin rash or itching	12	7	0.271	15	16	0.122	19	23	0.207
Swollen hands or feet	12	0	-	22	0	-	25	0	-
Spontaneous bruising	5	0	-	2	0	-	7	0	-
Hair loss	132	131	0.538	187	197	0.483	179	204	-
Painful or sensitive lips, painful swallowing, sore throat	58	20	0.232	73	26	0.131	73	23	0.188
Loss of appetite	71	28	0.341	63	30	0.276	63	25	0.393
Nausea	98	88	0.515	117	99	0.497	109	85	0.595
Vomiting	41	21	0.445	20	14	0.417	11	10	0.778
Diarrhoea	22	11	0.458	21	9	0.483	18	10	0.430
Dysuria	21	0	-	15	1	0.073	20	0	-
Loud breathing, wheezing	10	0	-	22	0	-	15	0	-
Cough	36	10	0.329	42	14	0.329	22	8	0.376
Painful breathing, dyspnoea	26	4	0.193	31	4	0.136	28	3	0.203
Watering and burning of the eyes	45	29	0.647	75	42	0.435	72	52	0.437
Changes in heart rhythm	20	2	0.018	27	1	0.092	20	0	-

**Table 3:** Representation of answers to questions by intensity of side effects.

	P2c	D2c	*p*	P3c	D3c	*p*	P4c	D4c	*p*
No side-effects reported									
None	824 (66.3)	975 (78.5)	<0.001	780 (62.8)	910 (73.3)	0.002	796 (64.1)	918 (73.9)	0.003
Reported side-effects									
Mild	169 (13.6)	142 (11.4)	0.126	151 (12.2)	187 (15.1)	0.050	165 (13.3)	188 (15.1)	0.221
Moderate	163 (13.1)	85 (6.8)	<0.001	195 (15.7)	73 (5.9)	<0.001	168 (13.5)	61 (4.9)	<0.001
Severe	86 (6.9)	40 (3.2)	<0.001	116 (9.3)	72 (5.8)	0.001	113 (9.0)	75 (6.0)	0.006
Total	418	267	<0.001	462	332	<0.001	446	324	<0.001

P2c = patient second cycle; D2c = physician second cycle; P3c = patient third cycle; D3c = physician third cycle; P4c = patient fourth cycle; D4c = physician fourth cycle. *p*-level of statistical significance of patients and physicians based on chi-square test. with zero degrees of freedom (assuming equal frequency of reporting side effects at. expected frequencies).

## Data Availability

The data that support the findings of this study are available from the corresponding author, upon reasonable request.
